# Hematological Parameters in Young Users of Heated Tobacco Products in Poland—A Case–Control Study

**DOI:** 10.3390/jcm14248779

**Published:** 2025-12-11

**Authors:** Małgorzata Znyk, Filip Raciborski, Beata Świątkowska, Dorota Kaleta

**Affiliations:** 1 Department of Population Studies, Department of Public Health, Medical University of Lodz, Zeligowskiego 7/9, 90-752 Lodz, Poland; beata.swiatkowska@umed.lodz.pl (B.Ś.); dorota.kaleta@umed.lodz.pl (D.K.); 2 Department of Environmental Hazard Prevention, Allergology and Immunology, Warsaw Medical University, Banacha 1a, 02-091 Warsaw, Poland; filip.raciborski@wum.edu.pl

**Keywords:** heated tobacco product, IQOS, health, blood counts, hematological parameters, morphological parameters

## Abstract

**Background/Objectives**: Young people are very susceptible to the marketing of technological devices and more frequently reach for heated tobacco products. There has been little research on how these products affect human health. The aim of the study was to assess the impact of heated tobacco use on hematological and biochemical parameters in young people. **Methods**: A case–control study was conducted in the years 2022–2025 among 200 healthy young individuals aged 18–30. The participants were divided into three groups, i.e., traditional cigarette smokers (DS), IQOS users (IQOS), and non-smokers (NS). Blood samples were collected from 111 subjects (38 IQOS, 28 DS and 45 NS), and morphological parameters were determined in the diagnostic laboratory at the Hospital of Brothers Hospitallers of St. John of God in Lodz. **Results**: Among the blood parameters analyzed, which did not follow a normal distribution, statistically significant differences in median values were identified between the NS, IQOS, and DS groups for uric acid (*p* < 0.01), hemoglobin (*p* < 0.05), mean corpuscular hemoglobin concentration (MCHC) (*p* < 0.05), and plateletcrit (PCT) (*p* < 0.01). Post hoc analysis revealed significant differences in uric acid levels between the NS and DS groups (4.3 vs. 5.2). For hemoglobin, statistically significant differences (*p* < 0.05) were found between the NS and IQOS groups (13.7 vs. 14.4). For MCHC, significant differences were also observed between the NS and IQOS groups (32.9 vs. 33.7). Among the multiple linear regression models, developed for variables with a normal distribution, only two models achieved an adjusted R^2^ above 0.4. In the model predicting red blood cells (RBC) levels, the adjusted R^2^ was 0.459. Two independent variables were significant, i.e., male sex (Beta = 0.703; *p* < 0.001) and DS compared to IQOS (Beta = −0.242; *p* < 0.01). The second model, predicting hematocrit levels, achieved an adjusted R^2^ of 0.458. Significant effects were noted for male sex (Beta = 0.700; *p* < 0.001) and DS versus IQOS (Beta = −0.235; *p* < 0.01). **Conclusions**: Monitoring hematological parameters can be used as an early predictor of morbidity in IQOS users. Therefore, there is a need for long-term studies that follow users over an extended period.

## 1. Introduction

In Poland, smoking is a widespread addiction that affects approximately 9 million people [1]. Among them, 27.1% of women and 30.8% of men declare themselves as daily (regular) smokers [2]. Most smokers begin smoking daily when they are very young (between 15 and 19 years of age) [3]. The PolNicoYouth study confirms that over 60% of Polish students and almost 50% of 15-year-olds have already smoked their first cigarette or used another nicotine product [2]. Tobacco-related diseases are reported to cause over 70,000 deaths in Poland annually [2].

According to the WHO (World Health Organization), in the global population, approximately 303 million people aged 15 and older currently use smokeless tobacco [4].

In recent years, several alternative products have been introduced to the market, including heated tobacco products (HTPs) [5]. At present, HTPs are strongly promoted by tobacco companies as a modern and less harmful alternative to regular cigarettes [6,7].

In 2014, Philip Morris’s IQOS (I-Quit-Ordinary-Smoking) was launched in Italy and Japan, quickly becoming the most widely used product [8]. Combustion in HTPs occurs at a temperature below 350 °C; in the case of IQOS, tobacco is heated to 201 °C [9,10]. In conventional cigarettes, the combustion takes place at a temperature of at least 600 °C [11,12]. These products were introduced to the market as an alternative to traditional cigarettes [13]. However, there is still no evidence that they are the less harmful option [14].

Heated tobacco products are sold in more than 40 countries, covering all six WHO regions. Distribution channels include flagship stores, supermarkets, shopping malls, as well as social, online and promotional events [15]. HTPs are classified as novel tobacco products (e.g., in Poland), smokeless tobacco products, tobacco products, or electronic cigarettes. In the United States, this category of products is defined and regulated as non-combustible cigarettes. In a few countries, HTPs are banned [15,16].

There are scarce data on the prevalence of HTP use [17]. In 2017, in Japan, 3.6% of the population aged 15–69 declared using IQOS in the previous 30 days [18]. In Italy, 1.4% of the population aged 15 and over have tried IQOS [19]. In the United Kingdom (UK), 1.7% of adults have used or tried heated tobacco products, with 13% of this group using them daily [18]. In the same year in Italy, 1.4% of the population aged 15 and over tried IQOS [20].

Due to the aggressive marketing activities of the tobacco industry, the number of individuals using various nicotine products has been growing rapidly. They are especially popular among young adults (aged 18–30 years). They are very susceptible to the marketing of technological devices and are increasingly reaching for HTPs [21]. Data show that new nicotine products are being used by young people who have never smoked, which represents a new trend in nicotine addiction [22].

Little is known about the impact of HTPs on the health of those who use them.

In recent years, independent studies have been conducted on heated tobacco products, showing a major health impact [10,22,23,24]. Data indicate that the chemical composition of aerosols released during the use of heated tobacco products may cause health effects similar to those associated with smoking traditional cigarettes [25]. Some studies prove that users of heated tobacco products have a lower risk of developing cardiovascular diseases, respiratory diseases, and cancer compared to smokers of traditional cigarettes [26,27]. However, there are few long-term studies conducted to assess the safety of these products on human health (including the risk of developing cancer) [28].

Such comparative analyses can contribute to a better understanding of the potential health implications of heated tobacco products and inform public health policies and smoking cessation strategies targeted at young populations.

The aim of the study was to assess the impact of heated tobacco use on blood counts (hematological and biochemical parameters) in young people. The results obtained in this group were compared with those of smokers and non-smokers.

## 2. Materials and Methods

### 2.1. Population and Study Design

This was a case–control study conducted at the Department of Hygiene and Epidemiology, Medical University of Lodz, in the years 2022–2025. The study ultimately recruited 200 healthy individuals aged 18–30. The participants were recruited from among students of Lodz universities, IQOS sales outlets, and post-secondary schools. Information about the recruitment procedure was posted on social media and the website of the Medical University of Lodz. Based on a short interview concerning health condition and smoking status, individuals who met the inclusion criteria and gave informed consent to participate in the study were assigned to one of the following groups: (a) smoking only traditional cigarettes (*n* = 65), (b) using only IQOS (*n* = 70), (c) non-smokers (*n* = 65).

The exclusion and inclusion criteria and a detailed description of the study are presented elsewhere [29].

IQOS users are defined as those who have never smoked or have not smoked for six months. Traditional cigarette smokers are those who for at least a year have smoked a minimum of five cigarettes a day and have not used other smoking substitutes. The study design assumed that each group had only one smoking habit. According to self-reported smoking habits, no concurrent smokers were identified. The study eligibility for each participant was initially assessed based on their self-reported smoking patterns or use of tobacco products and their self-reported health status. Depending on the self-reported data, each participant was assigned to one of the three groups.

In the first stage of the study, the participants completed a questionnaire and then underwent anthropometric measurements, blood pressure measurements, spirometry, and saliva sampling to assess cotinine levels. In the second stage, blood samples were collected from the study subjects at the Hospital of Brothers Hospitallers of St. John of God in Lodz.

The study was approved by the Bioethics Committee of the Medical University of Lodz (decision No. RNN/290/21/KE of 14 December 2021). Written informed consent to participate in the study was provided by all the subjects.

### 2.2. Questionnaire

The standardized questionnaire included data on socio-demographic characteristics (age, sex, place of residence, professional status, level of education, marital status). The questionnaire was adapted for the study based on the GATS (Global Adult Tobacco Survey) [30]. Its sections covered the following: smoking status (use of heated tobacco products, smoking), passive exposure to tobacco smoke, health status, and lifestyle (nutrition, physical activity, alcohol consumption, stress). The socio-demographic data included in the questionnaire were used for the purposes of this article.

Due to the relatively small number of study participants, place of residence was presented in three broad categories (village, city up to 200,000 residents, city above 200,000 residents). In Poland, approximately 40% of the population lives in rural areas. According to data from the Central Statistical Office, the average size of a population in cities with county rights (“cities with the poviat status”) is 186,488 inhabitants, which rounds to approximately 200,000. Therefore, this threshold reflects a meaningful demographic division in the Polish context.

### 2.3. Blood Parameter Measurement

Blood samples (20 mL) were taken from the study participants at the Hospital of the Brothers Hospitallers of St. John of God in Lodz, in accordance with the relevant guidelines on collecting samples for laboratory tests [31]. Blood was collected in the morning (between 7:00 and 10:00 AM), fasting, after a night’s sleep.

The obtained biological material was analyzed for hematological parameters, such as white blood cell (WBC) count, red blood cell (RBC) count, platelet count (PLT), monocyte count, plateletcrit (PCT), hemoglobin concentration (Hb, HGB), hematocrit (HCT), and erythrocyte indices, i.e., mean corpuscular volume (MCV), mean corpuscular hemoglobin concentration (MCHC), mean corpuscular hemoglobin (MCH), and red cell distribution width (RDW). Additionally, the following biochemical parameters were analyzed: total cholesterol, HDL (high-density lipoprotein), LDL (low-density lipoprotein), TG (triglycerides), glucose, fibrinogen, uric acid (UA), apolipoprotein A1 (Apo A1), apolipoprotein B (Apo B), and C-reactive protein (CRP). The whole procedure of sample collection and determination of blood parameters is described elsewhere [29].

Blood laboratory test results were obtained from 111 study subjects. Hematological and biochemical tests were performed by qualified laboratory diagnosticians, using a SYSMEX HN-550 analyzer (SYSMEX EUROPA SE, Norderstedt, Germany) and a ROCHE COBAS PURE analyzer (ROCHE, Warsaw, Poland), respectively.

All the subjects gave written informed consent to participate in the study. The study was funded by the National Science Center (NCN) (research project No. 2021/41/N/NZ7/00020).

### 2.4. Statistical Analysis

Data analysis was performed using statistical software IBM SPSS 29.0.0 (IBM, Armonk, NY, USA, IBM Corp.). Qualitative variables were analyzed by calculating the number (n) and percentage (%) of occurrences of each value. Quantitative variables were analyzed by calculating the median, mean, standard deviation (SD), as well as minimum (min) and maximum (max) values. The normality of the distribution of continuous variables was checked using the Shapiro–Wilk test. The result is presented in the online supplement (Table A1). Quantitative variables that did not follow a normal distribution (*p* < 0.05) were analyzed using the Kruskal–Wallis test (Table A2). Post hoc analysis following the Kruskal–Wallis test was conducted using Dunn’s test with Bonferroni correction. Quantitative variables with a normal distribution were analyzed using ANOVA. These analyses were performed at a significance level of 0.05; therefore, values of *p* < 0.05 were considered statistically significant.

## 3. Results

### 3.1. Characteristic of the Respondents

A total of 111 participants provided biological samples for the study. Among them, 45 individuals (40.5% of the sample) were never smokers (NS), 38 (34.2%) were IQOS users, and 28 (25.2%) were daily traditional cigarette smokers (DS). The study group included 31 men (27.9%) and 80 women (72.1%). The lowest proportion of men was observed in the NS group (13.3%), while the highest was in the DS group (46.4%). The mean age of the analyzed cohort was 21.2 years (SD = 2.5; min–max 18–29); the lowest mean age was observed in the NS group (20.8; SD = 2.4; min-max 19–24), and the highest in the DS group (21.7; SD = 2.5; min–max 18–29). The study participants started using IQOS at the age of 18.5 (SD = 2.3) and continued it for a mean period of 2.4 years (SD = 1.4). Whereas traditional cigarette smokers started smoking at the age of 16.3 (SD = 2.0) and continued it for a mean period of 4.5 years (SD = 3.0). The respondents most often used IQOS from six to ten times a day (39.5%). The mean number of cigarettes smoked by traditional smokers was 8.3 cigarettes (SD = 5.4) per day. Detailed group characteristics (NS, IQOS, DS) are presented in Table 1.

### 3.2. Blood Parameters

**Figure 1:**
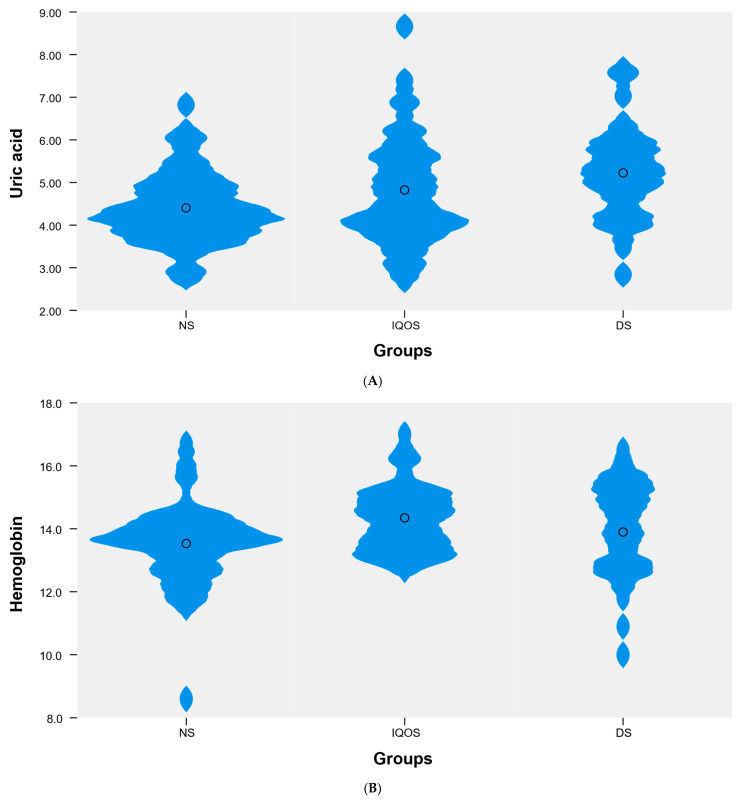

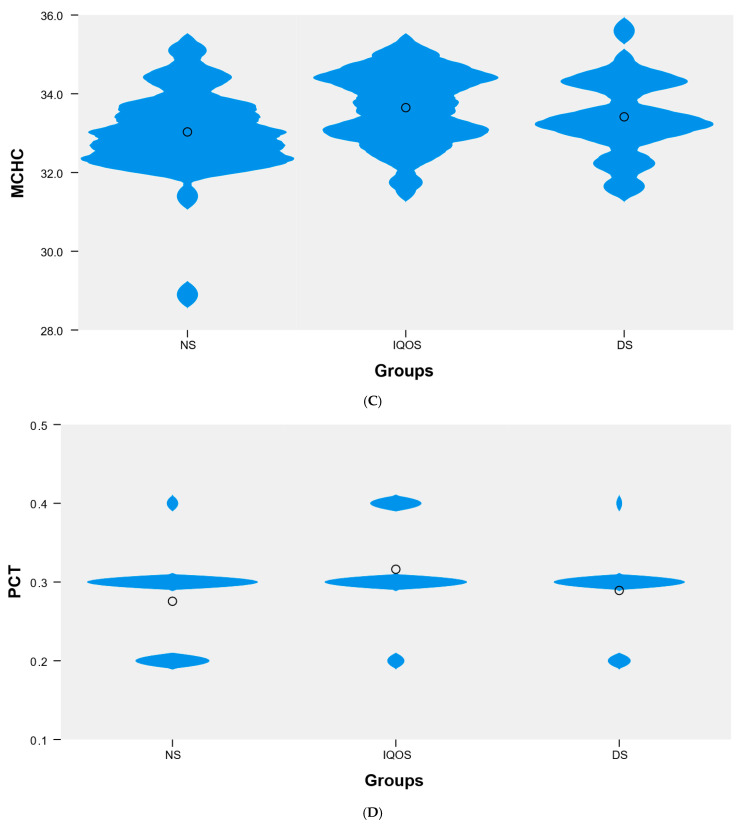
Violin plot showing the distribution of the variable (**A**) uric acid, (**B**) hemoglobin, (**C**) MCHC, and (**D**) PCT in the following groups: NS (*n* = 45), IQOS (*n* = 38), DS (*n* = 28).

Among the blood parameters analyzed, which did not follow a normal distribution, statistically significant differences in median values were identified between the NS, IQOS, and DS groups for uric acid (UA) (*p* < 0.01), hemoglobin (*p* < 0.05), MCHC (*p* < 0.05), and PCT (*p* < 0.01). Post hoc analysis revealed significant differences in uric acid levels between the NS and DS groups (4.3 vs. 5.2). For hemoglobin, statistically significant differences were found between the NS and IQOS groups (13.7 vs. 14.4). For MCHC, significant differences were also observed between the NS and IQOS groups (32.9 vs. 33.7). Detailed data are presented in Table 2, Figure 1. Otherwise the differences identified were not statistically significant.

As for the blood parameters with a normal distribution, no statistically significant (*p* > 0.05) differences in mean values were observed between the NS, IQOS, and DS groups. Detailed data are presented in Table 3.

Among the multiple linear regression models presented in Table 4, developed for variables with a normal distribution, only two models achieved an adjusted R^2^ above 0.4. In the model predicting RBC levels, the adjusted R^2^ was 0.459. Two independent variables were significant, i.e., male sex (Beta = 0.703; *p* < 0.001) and DS compared to IQOS (Beta = −0.242; *p* < 0.01). Age (*p* = 0.180) and BMI (*p* = 0.345) did not have a statistically significant effect. No significant effect of NS (vs. IQOS) was observed either. The second model, predicting hematocrit levels, achieved an adjusted R^2^ of 0.458. Significant effects were noted for male sex (Beta = 0.700; *p* < 0.001) and DS versus IQOS (Beta = −0.235; *p* < 0.01). Age (*p* = 0.484) and BMI (*p* = 0.799) were not statistically significant. Detailed data for the remaining models are presented in Table 4.

## 4. Discussion

Our study is one of the first designed to assess the health effects of IQOS use. So far in Poland, no studies have been conducted on young people that would analyze blood count parameters.

The results of our study indicate significant differences in median values between the three analyzed groups (IQOS, DS, NS) for uric acid, hemoglobin, mean hemoglobin concentration in red blood cells and platelet hematocrit.

Our study found statistically significant differences in uric acid (UA) levels between nonsmokers and traditional cigarette smokers (4.3 vs. 5.2). Levels of blood uric acid are increased in smokers compared to nonsmokers. Elevated levels of uric acid are associated with a greater risk of developing cardiovascular disease, gout, and hypertension. Research findings regarding levels of serum UA in smokers are inconsistent and partially gender-dependent [32]. Higher UA levels were observed in women with increased tobacco exposure. The risk of hyperuricemia increased in women with tobacco exposure after adjusting for confounding factors [33]. Female smokers have been shown to have higher serum UA concentrations compared to non-smoking women [34,35]. Higher serum UA concentrations were more common in men who smoked cigarettes concurrently, with a smoking history of more than 20 pack-years [35]. Other studies confirm higher UA concentrations in smokers of both sexes compared to non-smokers [36,37]. Also, a study conducted in Korea found that electronic cigarette use was associated with higher levels of blood uric acid. Similarly, people who used traditional cigarettes and e-cigarettes simultaneously showed higher levels of blood uric acid [38]. A study in Romania found an association between electronic cigarette use and increased levels of UA [39]. Other studies have reported significantly lower levels of uric acid in people using traditional cigarettes compared to non-smokers, regardless of sex [34,40,41,42]. Low levels of serum UA in smokers are associated with a higher incidence of lung cancer and chronic obstructive pulmonary disease [43].

Smoking also has serious negative effects on hematological parameters, which may be associated with an increased risk of developing polycythemia vera, cardiovascular diseases, atherosclerosis, and chronic obstructive pulmonary disease [44]. Individuals who smoke cigarettes have significantly higher levels of hematological parameters (HGB, HCT, RBC, MCHC, MCH, MCV, GR%, WBC) than other groups [45]. Smoking causes an increase in the number of neutrophils, leukocytes, monocytes and lymphocytes in the blood, hemoglobin, hematocrit and mean red blood cell volume [46].

Our study demonstrated statistically significant differences in hemoglobin between non-smokers and IQOS users (13.7 vs. 14.4). There are no other studies on this issue among the population using heated tobacco products; however, numerous papers on smokers of traditional cigarettes have been published [47,48,49]. In a study by Malenica et al., smokers had significantly higher levels of white blood cells, hemoglobin, MCV, and MCHC. Men who smoked cigarettes showed significant increases in red blood cell count, white blood cell count, hematocrit, hemoglobin, and MCHC compared to female smokers [44]. In a study by AlQahtana et al., it was found that smoking cigarettes or shisha was associated with an increase in hemoglobin levels [50]. In the study by Ahmed et al., Hb level, RBC count, neutrophils, WBC count, MCHC, RDW, MCH and PDW in smokers were significantly higher compared to the non-smoking group [51]. In another study, individuals who exclusively used e-cigarettes had higher mean concentrations of red blood cell hemoglobin compared to those who did not use e-cigarettes [52]. Increased HGB levels, by reducing blood velocity and creating polycythemia, may increase the risk of coronary vascular resistance, intravascular clots and thrombosis [53].

In our study, statistically significant differences were found for MCHC between non-smokers and IQOS users (32.9 vs. 33.7). In the study by Schmitt et al., smokers showed higher MCHC values, along with MCV, MCH, and hemoglobin, than former smokers and non-smokers [54]. These findings, however, are not confirmed by other studies [53,55]. Pankaj et al. found significantly lower MCHC values among smokers [55]. Similarly, Asif et al. observed a decrease in MCHC and MCH levels in smokers [53].

Tobacco smoking has a profound effect on platelet activation and morphology. It also impacts coagulation, hemostasis, and the healing cascade [56]. Smoking is associated with increased levels of PLT-dependent thrombin, which may induce a prothrombotic state [57]. Compared to non-smokers, smokers have been found to have elevated levels of thrombopoietin, which induces platelet production and causes an increase in PLT [58]. Chronically increased PLT activation is associated with inflammation and thrombosis, i.e., factors involved in the atherosclerotic process leading to cardiovascular disease [13]. In a study by Lyytinen et al., clot formation in PLT was significantly increased after exposure to HTP compared to no exposure [13]. In another study, PCT, PDW, and platelet count were significantly higher in smokers than in non-smokers [59]. Other studies have reported a slightly lower PLT in the group of non-smokers and electronic cigarette users than in the group of people using traditional cigarettes [52]. Passive smokers had statistically higher levels of PCT and PLT [45]. In a study by Tulgar et al., no significant differences in PCT and PLT count were found between non-smokers and smokers [60]. The platelet count may be low, indicating platelet abnormalities due to a failure of the bone marrow to respond to peripheral platelet demands [53]. In our study, no differences in medians were found between the three groups analyzed for PCT, despite the statistically significant result (*p* < 0.01).

Only two multiple linear regression models developed for normally distributed variables achieved adjusted R^2^ square values above 0.4 for RBC and hematocrit. In the model predicting RBC levels, the influence of the two independent variables, male gender and DS, was significant in relation to IQOS. Similarly, in the model predicting hematocrit levels, the influence of male gender and DS was significant in relation to IQOS. 

In the study by Manietta et al., analyses adjusted for gender, age, body mass index, and race/ethnicity showed that exclusive e-cigarette use was associated with significantly elevated red blood cell indices, i.e., hematocrit, hemoglobin, and mean corpuscular volume, in addition to elevated white blood cell counts (monocytes, lymphocytes, neutrophils) in the blood [52]. This observation supports previous evidence that combustible tobacco products drive an inflammatory response, potentially triggered by endothelial dysfunction, oxidative stress, and repeated exposure to toxic combustion byproducts [46].

In another multiple linear regression analysis, after adjusting for BMI, gender, age, and physical activity, active and heavy smokers, and groups of those smoking cigarettes for more than 15 years had the maximum positive significant correlation with HGB, HCT, MCH, MCV, and MCHC [45]. The reason for the increased hematocrit may be related to the mild hypoxic environment created by cigarette smoke, which may stimulate the production of more RBCs to compensate [52]. An increase in HTC and RBC levels increases the risk of cardiovascular disease as it puts a burden on the vascular system [45].

A study by Lakshmi et al. found that hematocrit and hemoglobin levels were significantly higher in smokers, while the RBC count increased significantly with higher smoking intensity [61]. Other studies confirm that smoking causes an increase in RBC count and elevated RBC parameters [39,46,62].

Abstinence from smoking for seven days and then 14 days resulted in lower RBC, hematocrit, and hemoglobin levels in both the older and younger cohorts, respectively [62]. These results show that RBC markers (hemoglobin, hematocrit, RBC count) respond to smoking status and are not affected by age [63]. In a study by Shakiba et al., cigarette smokers had significantly higher levels of WBC, HCT, HGB, RBC, MCH, MCV, MCHC and GR% than the other groups [45]. In another study, hematocrit and erythrocyte count did not differ between the three analyzed groups (smokers, non-smokers, ex-smokers) [54]. In the study by Aldosari et al., there were no differences in RBC count, HGB, and MCV between smokers and nonsmokers [64].

Hematological parameters are essential elements in the assessment of the disease status [65]. They play an important role in diagnosing infections, inflammation, anemia, and coagulation disorders. Hematological abnormalities are associated with coronary heart disease, oxidative damage, atherosclerosis, chronic obstructive pulmonary disease, and cancer [66,67]. These findings suggest that tobacco and HTP use may induce early hematological changes even in otherwise healthy young individuals. Longitudinal studies with larger cohorts are warranted to confirm these associations, evaluate their potential clinical relevance, and better understand the long-term health risks associated with different forms of tobacco consumption.

### 4.1. Limitations of the Study

This study has several limitations that should be considered. One of them is a small sample size. Nevertheless, it should be emphasized that this study represents one of the first investigations in Poland assessing the effects of IQOS-type product use on hematological parameters among young adults, with a sample size comparable to or exceeding that of many previous international studies of a similar design. Another limitation is the reliance on self-reported data. The participants in our study were divided into three groups based on their self-reported tobacco use habits, which may be subject to bias. The young age of the participants indicates a short period of use of heated tobacco products or traditional cigarettes, which could have influenced the study results. Using IQOS or traditional cigarettes may not have yet caused health effects (such as changes in hematological blood parameters). Blood collection and laboratory testing are also low among young people (56% of those enrolled in the study). Furthermore, the study’s single-point observation is a limitation. The study questionnaire did not include questions about workplace exposure factors. However, our study participants were primarily students so they were not exposed to workplace hazards. Even if some of them did work, in most cases they had part-time jobs. Additionally, the authors were unable to find similar studies in this area in the Polish population to provide a better comparison of results. 

These limitations highlight the necessity of larger, long-term studies to clarify the health risks associated with IQOS and other tobacco products. Future investigations should involve more diverse and representative samples, apply objective biomarkers of exposure, and include longitudinal follow-up to determine the causal and time-dependent effects of heated tobacco use on hematological and other physiological parameters.

### 4.2. Implications for the Future

The results of our study could serve as a basis for future research in Poland. Furthermore, the findings could inform changes in tobacco control policy and limit advertising of heated tobacco products as less harmful than traditional cigarettes. Our findings may also be useful for health policymakers in designing and implementing effective smoking prevention programs to protect the Polish population. Tobacco control policies should adopt a comprehensive, multisectoral approach that integrates education, regulation, and public awareness initiatives to address the challenges posed by both traditional and emerging tobacco products. A future understanding of the long-term health impacts of heated tobacco will be crucial to developing effective prevention strategies and protecting future generations from the harmful effects of nicotine.

## 5. Conclusions

Monitoring hematological parameters can be used as an early predictor of morbidity in IQOS users. Because HTPs are primarily targeted at young people, it is important to obtain clear evidence demonstrating the negative effects of their use. The results of our study contribute to the advancement of knowledge in this area by demonstrating readily available, measurable, and short-term changes in hematological profiles associated with heated tobacco use. These changes may reflect early biological responses to exposure and precede more serious health effects that may result from exposure. To fully understand the long-term impact of HTP consumption on overall health, particularly the hematological and cardiovascular systems, longitudinal studies with extended follow-up are urgently needed. Such studies would allow for a more accurate assessment of chronic risk, progression of subclinical changes, and potential reversibility after cessation of exposure.

## Figures and Tables

**Table 1:** Characteristics of the study group.

	NS	IQOS	DS	Total
Total (n)	45	38	28	111
Age (mean ± SD)	20.8 ± 2.4	21.2 ± 2.5	21.7 ± 2.5	21.2 ± 2.5
min-max	19–24	19–26	18–29	18–29
BMI (mean)	21.8	23.2	24.3	22.9
	*n* (%)	*n* (%)	*n* (%)	*n* (%)
Sex				
Female	39 (86.7)	26 (68.4)	15 (53.6)	80 (72.1)
Male	6 (13.3)	12 (31.6)	13 (46.4)	31 (27.9)
Professional status				
permanently employed person	2 (4.4)	4 (10.5)	3 (10.7)	9 (8.1)
a person working in their own company	0 (0)	2 (5.3)	0 (0)	2 (1.8)
part-time worker	12 (26.7)	6 (15.8)	8 (28.6)	26 (23.4)
student	43 (95.6)	32 (84.2)	24 (85.7)	99 (89.2)
Marital status				
single	35 (77.8)	28 (73.7)	22 (78.6)	85 (76.6)
informal, stable relationship	9 (20)	10 (26.3)	5 (17.9)	24 (21.6)
married	1 (2.2)	0 (0)	1 (3.6)	2 (1.8)
Education				
general or vocational secondary schools	21 (46.7)	15 (39.5)	7 (25)	43 (38.7)
post-secondary	20 (44.4)	21 (55.3)	15 (53.6)	56 (50.5)
higher	3 (6.7)	1 (2.6)	6 (21.4)	10 (9)
other	1 (2.2)	1 (2.6)	0 (0)	2 (1.8)
Place of residence				
city up to 200,000 residents	10 (22.2)	7 (18.4)	9 (32.1)	26 (23.4)
city above 200,000 residents	22 (48.9)	26 (68.4)	17 (60.7)	65 (58.6)
village	13 (28.9)	5 (13.2)	2 (7.1)	20 (18)
Frequency of IQOS use during the day				
5		10 (26.3)		
6–10		15 (39.5)		
11–20		13 (34.2)		
IQOS usage time (years) (mean ± SD)		2.4 ± 1.4		
IQOS initiation age (mean ± SD)		18.5 ± 2.3		
Smoking time (years) (mean ± SD)			4.5 ± 3.0	
Smoking initiation age (mean ± SD)			16.3 ± 2.0	
Number of cigarettes per day (mean ± SD)			8.3 ± 5.4	

Legend: min—minimum; max—maximum; n—number of respondents; %—percent; BMI—body mass index; DS—daily traditional cigarette smoker; NS—never smoker; IQOS—IQOS user; SD—standard deviation.

**Table 2:** Comparison of Blood Parameter Medians Across Three Groups (NS, IQOS, DS).

	Median				
	NS (*n* = 45)	IQOS (*n* = 38)	DS (*n* = 28)	Total (*n* = 111)	*p*-Value
Glucose	87.0	85.5	85.0	86.0	0.211
TG (Triglycerides)	76.0	73.5	69.5	74.0	0.751
CRPL2 (CRP, C-reactive protein)	0.8	0.8	0.6	0.7	0.519
Uric acid	4.3	4.6	5.2	4.5	**<0.01**
Fibrinogen	230.0	231.0	226.0	227.0	0.839
White blood cells (WBCs)	5.8	5.4	6.1	5.9	0.538
Lymphocyte count	2.1	2.0	2.1	2.1	0.849
Neutrophil count	2.9	3.0	3.0	2.9	0.595
Monocyte count	0.5	0.5	0.5	0.5	0.238
% Monocytes	8.3	8.2	8.6	8.4	0.423
Eosinophil count	0.1	0.1	0.2	0.1	0.264
% Eosinophils	2.1	2.1	2.8	2.4	0.355
Basophil count	0.0	0.0	0.0	0.0	0.178
% Basophils	0.5	0.6	0.5	0.5	0.322
Hemoglobin	13.7	14.4	14.0	13.9	**<0.05**
MCV (Mean Corpuscular Volume)	88.7	89.3	89.3	89.1	0.893
MCH (Mean Corpuscular Hemoglobin)	29.4	30.3	30.3	29.8	0.098
MCHC (Mean Corpuscular Hemoglobin Concentration)	32.9	33.7	33.4	33.2	**<0.05**
Red cell Distribution Width–Standard Deviation (RDW-SD)	40.8	40.6	41.9	40.8	0.578
Red cell Distribution Width–Coefficient of Variation (RDW-CV)	12.4	12.3	12.1	12.3	0.723
Platelets	249.0	283.0	263.5	267.0	0.054
Plateletcrit (PCT)	0.3	0.3	0.3	0.3	**<0.01**

Legend: NS—never smokers; DS—daily traditional cigarette smokers; IQOS—IQOS users.

**Table 3:** Comparison of Mean Blood Parameter Values Across Three Groups (NS, IQOS, DS).

	Mean				
	NS (*n* = 45)	IQOS (*n* = 38)	DS (*n* = 28)	Total (*n* = 111)	*p*-Value
Cholesterol	170.2	174.1	168.2	171.0	0.724
HDL	64.8	61.0	61.8	62.7	0.437
LDL	88.5	95.8	89.1	91.2	0.447
% Lymphocytes	36.9	35.3	34.0	35.6	0.213
% Neutrophils	51.6	52.8	53.4	52.5	0.607
Red blood cells (RBCs)	4.6	4.8	4.6	4.7	0.164
Hematocrit	40.9	42.6	41.5	41.7	0.090
Platelet Large Cell Ratio (P-LCR)	30.8	30.0	29.8	3.2	0.801
Platelet Distribution Width(PDW)	12.8	12.6	12.5	12.7	0.847
Mean Platelet Volume (MPV)	10.7	10.6	10.6	10.7	0.812

Legend: HDL—high-density lipoprotein; LDL—low-density lipoprotein; NS—never smoker; IQOS—IQOS user; DS—daily traditional cigarette smoker.

**Table 4:** Results of multivariate linear regression models. Dependent variable: selected blood parameters.

	Beta	t	*p*-Value	Adj. R^2^
**Dependent variable: cholesterol**				
Sex: male	−0.183	−1.797	0.075	0.037
BMI	0.248	2.431	**<0.05**	
Age (years)	−0.095	−0.949	0.345	
NS	−0.061	−0.555	0.580	
DS	−0.080	−0.743	0.459	
**Dependent variable: HDL**				
Sex: male	−0.400	−4.229	**<0.001**	0.169
BMI	−0.150	−1.586	0.116	
Age (years)	−0.010	−0.110	0.912	
NS	0.022	0.212	0.832	
DS	0.101	1.008	0.316	
**Dependent variable: LDL**				
Sex: male	−0.036	−0.358	0.721	0.049
BMI	0.302	2.980	**<0.01**	
Age (years)	−0.054	−0.541	0.590	
NS	−0.082	−0.754	0.453	
DS	−0.134	−1.246	0.216	
**Dependent variable: % Lymphocytes**				
Sex: male	0.130	1.282	0.203	0.036
BMI	−0.228	−2.218	**<0.05**	
Age (years)	0.017	0.166	0.868	
NS	0.099	0.904	0.368	
DS	−0.066	−0.607	0.545	
**Dependent variable: % Neutrophils**				
Sex: male	−0.223	−2.190	**<0.05**	0.034
BMI	0.203	1.970	0.052	
Age (years)	−0.059	−0.596	0.553	
NS	−0.086	−0.784	0.435	
DS	0.039	0.363	0.717	
**Dependent variable: Red blood cells (RBCs)**				
Sex: male	0.703	9.210	**<0.001**	0.459
BMI	0.073	0.949	0.345	
Age (years)	−0.101	−1.349	0.180	
NS	−0.056	−0.686	0.494	
DS	−0.242	−2.988	**<0.01**	
**Dependent variable: Hematocrit**				
Sex: male	0.700	9.169	**<0.001**	0.458
BMI	0.020	0.255	0.799	
Age (years)	−0.053	−0.703	0.484	
NS	−0.098	−1.196	0.234	
DS	−0.235	−2.897	**<0.01**	
**Dependent variable: MPV**				
Sex: male	−0.045	−0.421	0.675	−0.03
BMI	−0.068	−0.638	0.525	
Age (years)	−0.058	−0.553	0.581	
NS	0.034	0.300	0.765	
DS	0.022	0.197	0.845	
**Dependent variable: PDW**				
Sex: male	0.014	0.135	0.893	−0.034
BMI	−0.082	−0.765	0.446	
Age (years)	−0.053	−0.510	0.611	
NS	0.027	0.240	0.811	
DS	−0.001	−0.012	0.991	

Legend: BMI—body mass index; NS—never smokers; DS—daily traditional cigarette smokers; LDL—low-density lipoprotein; HDL—high-density lipoprotein; MPV—mean platelet volume; PDW—platelet distribution width; Adj.—adjusted; R^2^—R-squared coefficient of determination; Beta—regression coefficient.

## Data Availability

The datasets analyzed during the current study are available from the corresponding author upon reasonable request.

## Abbreviations

The following abbreviations are used in this manuscript:

WHO	World Health Organization
HTP	Heated tobacco product
IQOS	I-Quit-Ordinary-Smoking
UK	United Kingdom
BMI	body mass index
GATS	Global Adult Tobacco Survey
SD	Standard deviation
NS	Never smoker
DS	Daily traditional cigarette smoker
IQOS	IQOS user
UA	Uric acid
HCT	Hematocrit
HGB, Hb	Hemoglobin
RBCs	Red blood cells
MCH	Mean Corpuscular Hemoglobin
MCHC	Mean Corpuscular Hemoglobin Concentration
GR%	percentage of granulocytes
WBCs	White blood cells
MCV	Mean Corpuscular Volume
PLTs	Platelets
PCT	Plateletcrit
TGs	Triglycerides
CRPL2	C-reactive protein, CRP
RDW-CV	Red cell Distribution Width–Coefficient of Variation
RDW-SD	Red cell Distribution Width–Standard Deviation
HDL	High-density lipoprotein
LDL	Low-density lipoprotein
MPV	Mean platelet volume
PDW	Platelet distribution width
P-LCR	Platelet large cell ratio
Apo A1	Apolipoprotein A1
Apo B	Apolipoprotein B

## Appendix A

**Table A1:** Shapiro–Wilk Test for Normality.

	W Statistic	df	*p*-Value
Glucose	0.963	111	0.004
Cholesterol	0.983	111	**0.161**
HDL	0.978	111	**0.063**
LDL	0.984	111	**0.193**
TG (Triglycerides)	0.861	111	0.000
CRPL2 (C-reactive protein, CRP)	0.439	111	0.000
Uric acid	0.965	111	0.005
Fibrinogen	0.880	110	0.000
ApoA1 (Apolipoprotein A1)	0.979	110	**0.077**
ApoB (Apolipoprotein B)	0.978	110	**0.062**
White blood cells (WBCs)	0.954	111	0.001
Lymphocyte count	0.970	111	0.012
% Lymphocytes	0.985	110	**0.264**
Neutrophil count	0.892	111	0.000
% Neutrophils	0.983	110	**0.173**
Monocyte count	0.972	111	0.018
% Monocytes	0.956	110	0.001
Eosinophil count	0.853	111	0.000
% Eosinophils	0.822	110	0.000
Basophil count	0.917	111	0.000
% Basophils	0.945	110	0.000
Red blood cells (RBCs)	0.979	111	**0.082**
Hemoglobin	0.974	111	0.030
Hematocrit	0.977	111	**0.053**
MCV (Mean Corpuscular Volume)	0.957	111	0.001
MCH (Mean Corpuscular Hemoglobin)	0.906	111	0.000
MCHC (Mean Corpuscular Hemoglobin Concentration)	0.961	111	0.003
RDW-SD (Red cell Distribution Width-Standard Deviation)	0.967	111	0.008
RDW-CV (Red cell Distribution Width-Coefficient of Variation)	0.850	111	0.000
Platelets	0.969	111	0.011
MPV (Mean Platelet Volume)	0.992	110	**0.803**
PCT (Plateletcrit)	0.712	110	0.000
PDW (Platelet Distribution Width)	0.987	110	**0.380**
P-LCR (Platelet Large Cell Ratio)	0.994	110	**0.927**

**Table A2:** Kruskal–Wallis Test Results for Groups: NS, IQOS and DS.

	Ranks			
	NS (*n* = 45)	IQOS (*n* = 38)	DS (*n* = 28)	*p*-Value
Glucose	62.5	52.4	50.5	0.211
TG (Triglycerides)	54.1	59.2	54.8	0.751
CRPL2 (C-reactive protein, CRP)	60.0	54.6	51.6	0.519
Uric acid	46.6	56.4	70.6	**<0.01**
Fibrinogen	55.2	57.7	53.0	0.839
White blood cells (WBCs)	54.5	53.5	61.8	0.538
Lymphocyte count	58.0	54.1	55.3	0.849
Neutrophil count	53.3	55.4	61.1	0.595
Monocyte count	52.3	53.8	64.8	0.238
% Monocytes	53.6	52.8	62.5	0.423
Eosinophil count	51.9	54.7	64.3	0.264
% Eosinophils	52.0	54.3	63.0	0.355
Basophil count	49.3	60.6	60.6	0.178
% Basophils	50.4	60.9	56.4	0.322
Hemoglobin	46.7	66.2	57.2	**<0.05**
MCV (Mean Corpuscular Volume)	54.2	57.1	57.4	0.893
MCH (Mean Corpuscular Hemoglobin)	48.1	62.1	60.4	0.098
MCHC (Mean Corpuscular Hemoglobin Concentration)	46.2	66.1	58.1	**<0.05**
RDW-SD (Red cell Distribution Width-Standard Deviation)	55.4	52.9	61.2	0.578
RDW-CV (Red cell Distribution Width-Coefficient of Variation)	58.8	53.2	55.3	0.723
Platelets	48.5	65.6	55.0	0.054
PCT (Plateletcrit)	47.3	66.5	54.1	**<0.01**
